# Bone Mineral Content Estimation in People Living with HIV: Prediction and Validation of Sex-Specific Anthropometric Models

**DOI:** 10.3390/ijerph191912336

**Published:** 2022-09-28

**Authors:** Igor Massari Correia, Anderson Marliere Navarro, Jéssica Fernanda Corrêa Cordeiro, Euripedes Barsanulfo Gonçalves Gomide, Lisa Fernanda Mazzonetto, Alcivandro de Sousa Oliveira, Emerson Sebastião, Bruno Augusto Aguilar, Denise de Andrade, Dalmo Roberto Lopes Machado, André Pereira dos Santos

**Affiliations:** 1 School of Physical Education and Sport of Ribeirao Preto, University of Sao Paulo at Ribeirao Preto, Sao Paulo 14040-900, Brazil; 2 Faculty of Medicine, University of Sao Paulo at Ribeirao Preto, Sao Paulo 14049-900, Brazil; 3 College of Nursing, University of Sao Paulo at Ribeirao Preto, Sao Paulo 14040-902, Brazil; 4 Anthropometry, Training and Sport Study and Research Group, School of Physical Education and Sport of Ribeirao Preto, University of Sao Paulo at Ribeirao Preto, Sao Paulo 14040-900, Brazil; 5 Health and Exercise Research Group, Department of Kinesiology and Physical Education, Northern Illinois University, Dekalb, IL 60115, USA; 6 Department, Human Exposome and Infectious Diseases Network (HEID), Ribeirao Preto, Sao Paulo 14040-902, Brazil

**Keywords:** acquired immunodeficiency syndrome, bone density, osteoporosis, aging

## Abstract

People living with HIV (PWH) experience an accelerated reduction in bone mineral content (BMC), and a high risk of osteopenia and osteoporosis. Anthropometry is an accurate and low-cost method that can be used to monitor changes in body composition in PWH. To date, no studies have used anthropometry to estimate BMC in PWH. To propose and validate sex-specific anthropometric models to predict BMC in PWH. This cross-sectional study enrolled 104 PWH (64 males) aged >18 years at a local university hospital. BMC was measured using dual energy X-ray absorptiometry (DXA). Anthropometric measures were collected. We used linear regression analysis to generate the models. Cross-validations were conducted using the “leave one out”, from the predicted residual error sum of squares (PRESS) method. Bland–Altman plots were used to explore distributions of errors. We proposed models with high coefficient of determination and reduced standard error of estimate for males (r^2^ = 0.70; SEE = 199.97 g; Q^2^_PRESS_ = 0.67; SEE_PRESS_ = 208.65 g) and females (r^2^ = 0.65; SEE = 220.96 g; Q^2^_PRESS_ = 0.62; SEE_PRESS_ = 221.90 g). Our anthropometric predictive models for BMC are valid, practical, and a low-cost alternative to monitoring bone health in PWH.

## 1. Introduction

Delay in the diagnosis and non-adherence to adequate treatment for the human immunodeficiency virus (HIV) leads to the development of the acquired immune deficiency syndrome (Aids) [1]. According to the Joint United Nations Program on HIV/AIDS (UNAIDS), approximately 36 million people living with HIV (PWH) have died from Aids-related illnesses between 1980 and 2020 [1]. Antiretroviral therapy (ART) is the standard treatment for HIV control and contributes positively to patient’s life by prolonging life expectancy, and decreasing the frequency of hospital admissions [2]. However, exposure to HIV and prolonged use of ART are associated with reduced bone mineral density area (aBMD), which increases the risk of osteopenia and osteoporosis in this population [3,4].

Osteoporosis is a systemic skeletal disease, and therefore, all bones can be affected by this condition. However, there are certain parts (e.g., spine, hip, and wrist) where it can cause a higher negative impact on the social life of those who have it. A study showed that the likelihood of developing osteoporosis is 2.8 times greater in the lumbar spine and 3.4 times greater in the hip in PWH on ART compared to PWH who do not undergo this type of therapy [5]. Osteoporosis and fracture risk varies according to sex, ethnicity, age, time of diagnosis, HIV viral load, exposure, and type of ART composition; CD4+ cell count, physical activity level, amongst others [6]. To this end, some effective means to prevent osteopenia, osteoporosis, and some of its undesirable consequences (i.e., fractures) include, but are not limited to, regular physical activity and balanced nutrition.

The importance of a personalized monitoring bone mass goes beyond predicting the risk of bone fractures: hypocalcemia, hyperphosphatemia, secondary hyperparathyroidism, decreased vitamin D, vascular calcification, decreased renal function, and inflammatory bowel disease have a relationship with decreased bone mass [7,8]. There is an inverse correlation between coronary artery calcification and bone mass [9]. In addition, bone mass is further positively associated with the amount of muscle mass in the body, which transposes to other aspects such as muscle strength and functionality, and that may be taking into consideration during the clinical analysis of the patient [10]. Other aspects such as frailty, malnutrition, and nutritional education are factors that can influence bone mass. Thus, a screening assessing risk factors such as low bone mass, including bone mineral content (BMC), frailty, and malnutrition are important factors to prevent the risk of bone fracture and other health deficits resulting from it [11,12,13].

Bone densitometry is a method capable of detecting changes in bone mineralization. aBMD is the ratio of BMC to area (g/cm^2^) and can be measured by several imaging methods, with the dual energy X-ray absorptiometry (DXA) being considered the reference method [14]. Reduced aBMD values are observed in people with a decrease in BMC [14]. In parallel, anthropometry was developed with the aim of providing the monitoring of body changes in an objective, safe, accurate, and low-cost manner. Adopting it as a means of predicting BMC in PWH would be important, especially for countries where financial resources are limited, providing an alternative to monitor PWH bone mass in countries that cannot afford high-cost equipment, such as DXA. This can also help other countries to reduce spending on the acquisition and maintenance of equipment for monitoring bone mass [14]. However, to the best of our knowledge, there are no studies in the scientific literature that have used anthropometry to estimate BMC in adult individuals living with HIV. Thus, this study aimed to propose and validate anthropometric models to predict BMC in PWH specific for sex.

## 2. Materials and Methods

This cross-sectional study recruited and enrolled PWH on ART from the University Hospital, School of Medicine at Ribeirão Preto, University of São Paulo, Brazil (HC–FMRP-USP/UETDI). This manuscript followed the guidelines from The Strengthening the Reporting of Observational Studies in Epidemiology (STROBE). Participants were recruited between November 2013 and November 2014. The inclusion criteria for the study were: diagnosis of HIV, aged 18 to 59 years, and at least six months on ART. The exclusion criteria included current treatment for opportunistic diseases (e.g., pneumocystosis, histoplasmosis, and tuberculosis) or cancer, use of medications with expected body composition alterations (i.e., testosterone, growth hormone, and insulin growth factor 1), being pregnant, complete or partial loss of a limb, or engaged in a supervised exercise program in the six months prior to the beginning of the study. From a total population of 1298 PWH in treatment at HC–FMRP-USP/UETDI during the time of data collection, and to achieve an accuracy of 90% with a standard error of estimate (maximum 400 g) for BMC, a sample size of 100 to 110 participants was recommended. The Power and Sample Size Program^®^ version 3.043 was adopted for sample size calculation. The study protocol was reviewed and approved by the Ethics Review Board of the School of Medicine of the University Hospital of Ribeirão Preto, University of São Paulo, Brazil, (process number: 7082/2011), in compliance with human subjects guidelines from the Resolution of the National Council of Health (CNS) 466/12 and the Declaration of Helsinki. All subjects signed an informed consent prior to data collection.

### 2.1. Procedures

BMC was assessed using a DXA Scanner Hologic^®^ (Discovery CI/WI, software version 11.2, Bedford, MA, USA), which was operated by a trained technician following standard procedures [15,16]. We used the Total-BMC in grams (g) (BMC_DXA_) as the dependent variable, predicted by potential independent variables that included anthropometric measurements (i.e., body mass, height, skinfolds, and body circumferences). We further collected additional predictor variables which were taken from the medical records and interview with each participant. This included: age (years), self-reported race/ethnicity (White, Black, Asian, or Pardo Brazilians) [17], formal education (years), time of HIV diagnosis (years since the diagnosis), time of ART (years since the beginning of treatment), and type of ART (if the patient uses protease inhibitor (PI) or not).

### 2.2. Anthropometric Assessment

We collected anthropometric measures from the participants in order to establish anthropometric predictive models for BMC (BMC_Mod_Anthr_). The procedures for anthropometric assessment including body mass, height, body mass index (BMI) (body weight (kg)/height (m)^2^), skinfold thickness (SK), and body circumferences based on scientific works and reference manuals to conduct the anthropometric assessment [18]. The body circumferences of right arm extended, right arm contracted, left thigh (proximal), and left medial calf (largest diameter) were corrected for the corresponding segment of subcutaneous adipose tissue thickness [19]. We corrected the body circumferences measurements using skinfold thickness measurements to control for the influence of subcutaneous adipose tissue on our predictive models [20]. To ensure the quality of the data we took three precautionary steps. First, all the anthropometric measurements listed above were collected by the same evaluator at three-time points, and the median values were used [18]. In addition, the measurements were conducted with 6 patients, and they were re-tested an hour later to confirm accuracy. Finally, the technical error of measurement (TEM) was calculated. TEM values were assessed for body circumferences (TEM ≤ 0.71 cm) and skinfold thickness (TEM ≤ 0.60 mm), ensuring the reliability of the measurements within established limits [21].

### 2.3. Statistical Analysis

To validate and ensure the integrity of the data collected, double data entry technique (using Microsoft Excel^®^) was adopted. This helped ensure error-free data during the tabulation process. Additionally, exploratory analysis was used to investigate potential outliers. Descriptive analysis (i.e., measures of central tendency and confidence interval (95% CI)) was used for sample characterization grouped by sex. The differences between sex were calculated based on their characterization, body composition, and anthropometric variables using independent *t* test. Of note, we adopt parametric statistic procedures considering the central limit theorem [22]. Considering the potential influence of sex in body composition, we considered male and female groups separately in order to develop the anthropometric models to predict BMC_DXA_.

Principal component analysis was used to reduce the number of predictor variables. A total of 40 variables were created for the additional predictor to investigate if there was an effect on the models, for example, age, sex, and race/ethnicity were included in these variables that can modify the models. The Eigenvalues (>1) and highest extraction value (>0.90) were adopted as criteria for not exclusion of variables [23]. Five variables remaining after varimax rotations: body weight, BMI, right arm flexed corrected circumference (RAFCC), right arm flexed circumference (RAFC), and abdomen circumference (AC). We further used stepwise linear regression, adopting limits of VIF < 7.0, and Eigenvalues >  0.4 [23]. Bland–Altman plots were used to investigate limits of agreements between BMC measured (BMC_DXA_) and predicted (BMC_Mod_Anthr_), with a 95% CI [24]. All statistical analyses were conducted using SPSS^®^ version 20.0 (IBM Corporation, Armonk, NY, USA) with a significance level of α = 0.05. Cross-validation of the models were conducted using the “leave one out”, from the predicted residual error sum of squares (PRESS) method, to measure the efficiency of each BMC_Mod_Anthr_ generated [23,25]. The coefficients Q^2^ _PRESS_ and error of estimate (S) _PRESS_ were expressed for all BMC_Mod_Anthr_ separated by sex. The PRESS analysis was conducted using Minitab^®^ software version 17 (State College, PA, USA: Minitab, Inc. [www.minitab.com] accessed on 11 May 2022).

## 3. Results

Detailed characteristics of the sample overall and separated by sex are displayed in Table 1. Briefly, the average age of the male participants was 45.3 years and female 44.0 years. We observed homogeneity of participants between age, time of HIV diagnosis, time of exposure to ART treatment, protease inhibitors use, formal education, and self-reported race/ethnicity between males and females.

For body composition measured through DXA, males had a total BMC value higher than females (*p* ≤ 0.001). Regarding anthropometric assessment, males presented with higher values of height, body weight, left forearm, left wrist, right arm extended corrected, right arm contracted corrected, right forearm, right wrist, left ankle, right medial calf corrected, right ankle, shoulder, breast, and waist. In addition, lower values for BMI, left thigh, right thigh, hip, and skinfold thickness of triceps, thigh, and medial calf were also observed for the male participants.

### 3.1. Predictive Models to Estimate BMC_DXA_

Multiple stepwise linear regression BMC_Mod_Anthr_ models were proposed for males and females. Details of each BMC_Mod_Anthr_, validation, and errors estimates from BMC_DXA_ and BMC_Mod_Anthr_ are presented below. Table 2 displays three BMC_Mod_Anthr_ models for males. After conducting multiple stepwise linear regressions, we observed variations of the adjusted r^2^ value between 0.55 and 0.70, SEE (g) between 199.97 and 238.74, and 95% CI between 184.10 and 245.87. The BMC_Mod_Anthr_ 2 and 3 revealed the highest coefficient of determination (i.e., 0.67 and 0.70), and lowest SEE (g) (i.e., 208.74 and 199.97). The referred models are described below.

**BMC_Mod_Anthr 2_** = 1164.90 + (Body weight _[kg]_ × 42.15) + (BMI _[kg/m^2^]_ × −78.42)

**BMC_Mod_Anthr 3_** = 557.55 + (Body weight _[kg]_ × 40.11) + (BMI _[kg/m^2^]_ × −86.93) + (RAFCC _[cm]_ × 34.77)

**Figure 1:**
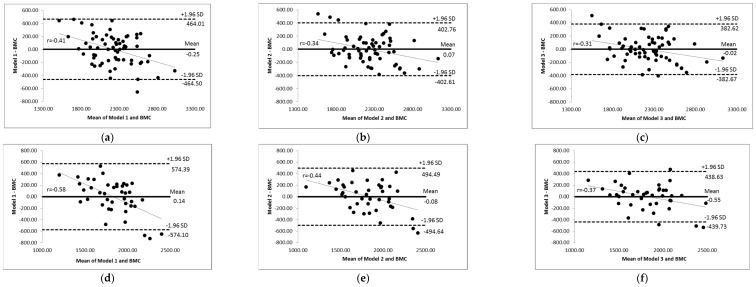
Bland–Altman plots for bone mineral content using DXA (BMC_DXA_) and bone mineral content using anthropometric predictive models (BMC_Mod_Anthr_) for males (**a**–**c**) and females (**d**–**f**) living with HI. (**a**) Bland–Altman plot (BMCMod_Anthr 1) for males. (**b**) Bland–Altman plot (BMCMod_Anthr 2) for males. (**c**) Bland–Altman plot (BMCMod_Anthr 3) for males. (**d**) Bland–Altman plot (BMCMod_Anthr 1) for females. (**e**) Bland–Altman plot (BMCMod_Anthr 2) for females. (**f**) Bland–Altman plot (BMCMod_Anthr 3) for females.

Figure 1 displays the Bland–Altman plots for BMC_DXA_ and BMC_Mod_Anthr_ for males (a, b, and c). The agreement between the BMC_DXA_ and BMC_Mod_Anthr_ was accurate, since the models exhibited no significant bias (−0.25 until +0.07), with limits of agreement reduced especially for BMC_Mod_Anthr_ 2 (−402.61 and  +402.76) and BMC_Mod_Anthr_ 3 (−382.67 and  +382.62). In addition, it was observed that there was a small tendency for predictive models to underestimate the BMC_DXA_ when the BMC values were higher, and to overestimate when the BMC values were lower. We observed a weak association between the mean differences and the mean of the two measurements. However, there was an accurate BMC_Mod_Anthr_ estimate of the BMC_DXA_, since the extreme values of BMC were rarely outside the limits of agreement.

Table 2 shows three BMC_Mod_Anthr_ for females. After conducting multiple stepwise linear regressions, we observed variations of the adjusted r^2^ value between 0.33 and 0.65, SEE (g) between 220.96 and 300.71, and 95% CI between 179.47 and 310.20. The BMC_Mod_Anthr_ 3 model presented the best coefficient of determination of 0.65 and the lowest SEE (g) of 220.96. The referred model is described below.

**BMC_Mod_Anthr 3_** = 2881.94 + (Body weight _[kg]_ × 58.94) + (AC _[cm]_ × −34.82) + (RAFC _[cm]_ × −53.54)

Figure 1 shows the Bland–Altman plots for BMC_DXA_ and BMC_Mod_Anthr_ for females (d, e, and f). The agreement between the BMC_DXA_ and BMC_Mod_Anthr_ was considered accurate, since the models exhibited non-significant bias (−0.55 until +0.14), with limits of agreement reduced especially for the BMC_Mod_Anthr_ 3 (−439.73 and  +438.63). In addition, it was found that there was a small tendency for predictive models to underestimate the BMC_DXA_ when the BMC values were higher, and to overestimate when the BMC values were lower. We observed a moderate association between the mean differences and the mean of two measurements. However, BMC_Mod_Anthr_ was able to accurately predict BMC_DXA_, since the extreme values of BMC were rarely outside the limits of agreement.

### 3.2. Validation of Predictive Models to Predict BMC_DXA_

For the BMC_Mod_Anthr_ 2 and 3 for males (Table 2), we observed high adjusted Q^2^ _PRESS_ values (0.64 and 0.67) with small S _PRESS_ (g) values (215.07 and 208.25). For the BMC_Mod_Anthr_ 3 for female, we also observed a high adjusted Q^2^ _PRESS_ value (0.62), and a small S _PRESS_ (g) value (221.90). Collectively, the short intervals for the limits of agreement observed in each predictive model by Bland–Altman plots and the adjusted Q^2^ _PRESS_ values support the accuracy of these sex-specific predictive models for estimating BMC_DXA_ and identifying bone alterations in PWH.

## 4. Discussion

This study sought to propose and validate predictive models to identify BMC in PWH through anthropometric measurements using DXA as the criterion measure. This is important because anthropometric measurements are accurate, low-cost, and easy-to-use. To ensure global dissemination and accessibility, BMC based on our predictive models can be found in an excel file in the following link “http://posgraduacao.eerp.usp.br/files/BMC.xlsx (accessed on 15 September 2022)”. Especially in low- and middle-income countries, our predictive models may help in enhancing monitoring and assessments in this area, which may translate into improvements in the prevalence, incidence, risk factors, pathogenesis, treatment, and prevention of bone changes (early identification in the reduction of BMC and appropriate treatment). In addition, the viable identification of changes in BMC will contribute to development of interventions that lead to better health outcomes, including general well-being among PWH, as well as reduced costs due to hospitalizations.

Our study is important not only in the context of Brazil, but also other countries with limited resources available to develop such studies [26]. A 2017 literature review suggested that the difficulty in treatments related to bone health of the population in South Africa was mainly due to the lack of equipment for aBMD assessment with an ideal cost–benefit ratio for the country’s economic situation [26]. The maintenance of DXA requires not only structural but also financial resources. Furthermore, such equipment is scarce in regions where HIV is more prevalent (i.e., low- and middle-income countries). Our anthropometric models may help in the control of osteoporosis due to HIV infection and side effects of ART, making it possible to improve the well-being of PWH in patients living in those countries.

We observed differences in body composition between males and females, mainly in the BMC values, which reinforces the need to develop specific models for BMC_Mod_Anthr_. In addition, considering the homogeneity between sexes for the variables of age, time of diagnosis for HIV, time of exposure to ART, protease inhibitors use, and race/ethnicity, the differences observed in BMC_DXA_ values are probably due to the specific characteristics of each sex [27,28,29]. The anthropometric differences between males and females PWH, observed specially in body circumferences and skinfold thickness are those expected and reported in scientific literature [16,30].

Bone alterations are perceptible in PWH, exposing them to a higher risk for fractures in comparison with seronegative individuals. The prevalence of fractures (spine, hip, or wrist) in PWH compared to seronegative persons is 2.87 per 100 people [23]. PWH demonstrate a reduction in bone mass compared to their counterparts who are seronegative. However, PWH on ART treatment present with lower aBMD and BMC values compared to seronegative individuals, and PWH that are not on ART [5].

Overall, bone mass degradation begins around the age of 35 years old with a reduction rate of 0.5 to 1% per year [31]. However, PWH demonstrate age-related fracture rates that may be higher than those in seronegative persons [32]. PWH are more impacted by hormonal, bone, and muscle changes, which can lead to important functional damage. Middle-aged PWH muscle mass is similar to seronegative individuals 10 to 25 years older, which demonstrates a faster loss of functionality in this population when compared to the seronegative population [33]. ART is associated with mitochondrial dysfunctions that are related to the aging process and bone mass decrease [34]. The differences between the components of ART can cause different consequences, one example is the use of protease inhibitors (PIs), that can stimulate the development of hyperparathyroidism which increases bone reabsorption and bone mass loss [5].

Osteoporotic fractures negatively impact quality of life and decrease life expectancy. There is evidence suggesting that hip fractures increase mortality rate from 12% to 20% in the two years following a fracture and more than half of the individuals who survive after hip fracture cannot live independently [35]. Therefore, PWH have a higher risk of suffering such consequences compared to seronegative individuals. We proposed accurate anthropometric models, as indicated by the adjusted r^2^ values. To the best of our knowledge, the present study is the first to propose and validate these models to identify and monitor bone changes in adults with HIV, adopting a simplified, safe, and accessible method (i.e., anthropometry). There are few studies in the scientific literature who adopt anthropometry to assess body composition alterations in PWH [14,30,36]. One study conducted by Lima et al. (2016) proposed anthropometric models to identify bone alterations in children and adolescents living with HIV. In this work, the coefficient of determination of the BMC was 0.94, and our study showed lower values for the prediction of BMC [14]. However, there were several differences between the two studies, including environmental aspects, life habits, and time on ART. Furthermore, in our study we used BMI rather than height and weight which may have accounted for some of the differences. Overall, our findings reinforce the feasibility and the reliability of anthropometric methods for bone mass assessment and add to the current body of knowledge by proposing and validating sex-specific models for adults living with HIV.

The BMC anthropometric models for females included body weight, AC, and RAFC, while for males, it included body weight, BMI, and RAFCC. Females under 50 years of age had a positive association between BMC and variables related to body fat mass. On the other hand, males showed a positive association between BMC and lean body mass but without a significant association with body fat mass [36,37]. This may be one of the reasons for the difference between the sexes in the precision of predictive models. The independent variables were different according to sex, with a greater importance of body fat for females (AC and RAFC) and lean mass for males (RAFCC) [16]. The variable body weight was included for both male and female models, which is associated with bone mass, as the total body weight imposes mechanical load on the bones [38,39].

Our findings should be interpreted with caution due to some limitations. We did not control the dosage of ART, other medication therapy (e.g., levels and consumption of vitamin D), diet, physical activity level, menopause period, or race/ethnicity influence. However, even though we did not assess the impact of ART, vitamin D levels, or steroid synthesis on BMC, the class of antiretroviral medication (protease inhibitor) was not found to be a relevant variable to be included in the prediction models after principal component analysis. Menopause is part of the aging process which is associated with hormonal, metabolic, and bone changes [39,40]. The majority of participants in our study self-identified themselves as White. Furthermore, age and race/ethnicity variables were not included in our prediction models after principal component analysis. However, aware of the importance of these variables in our study, we were careful to consider them in the proposition of our models. Our first attempt was to disregard the results of the principal components analysis (referring to age and race/ethnicity) and in the stepwise linear regression analysis include them. The same process was used to the variables maintained from principal component analysis, such as body weight, BMI, RAFCC, RAFC, and AC. For both sexes, exactly the same models presented in Table 2 were found. For our second attempt, we sought to “force” the age and race/ethnicity variables into the models. In this sense, we opted for the “enter” method of linear regression analysis. For this attempt, in both sexes it was observed that: (a) there was an increase in the number of independent variables; (b) reduction in the model’s predictive power; (c) increase in the standard error of estimate; and (d) unbalanced VIF values and Eigenvalues that inflate the model. Thus, we decided to follow the statistical assumptions for the principal components analysis and generation of predictive models [23,25]. To this end, the models presented in the manuscript are the most appropriate proposal for the investigated sample. Our models were generated based in a narrow age range excluding older adults and in a mixed race sample. Thus, it might not be appropriate for people out of this age range and race/ethnicity different until external validation across other samples. In addition, we did not exclude PWH with chronic metabolic diseases, including those associated with bone loss. These conditions are often experienced by PWH on ART treatment. Even though some chronic metabolic diseases may influence body composition, our goal was to study the most typical group of PWH. However, we understand that the presence of chronic metabolic diseases could have influenced our predictive models.

Another point to be observed is the fact that the result of the anthropometric measurements is dependent on the way the evaluator performs the measurements, which may be different among evaluators, and this can be a factor that can interfere with the results of the models. To minimize such a possibility, a single experienced evaluator conducted all the measurements in our study. Despite these limitations we were able to establish novel, important, and preliminary valid evidence of anthropometric models to predict BMC in PWH.

The scientific literature describes other alternative methods to predict the risk of bone fractures: the Fracture Risk Assessment Tool (FRAX), radiofrequency echographic multi-spectrometry (REMS), low-frequency quantitative ultrasound measurement (LFQUM), and machine learning models for prediction of osteoporosis from clinical health examination data (MLMPO). These models present good results for predicting the risk of bone fractures; however, none of them have any specificity for the population of our study. The REMS and LFQUM models are methods that provide accurate aBMD values, however, they require specific devices that may be considered expensive for low and middle income countries [41,42]. Parallel, LFQUM uses sound waves to measure bone density. It is quick and painless. And it does not use potentially harmful radiation such as X-rays. One downside of ultrasound is that it cannot measure the density of the bones in the hip and spine [43]. FRAX is a free online tool that estimates the risk of having a hip or other major fracture in the next 10 years. FRAX is administered by a health care provider, and it is applied only for people who meet certain conditions (e.g., “having low bone density (osteopenia)”, “not currently taking osteoporosis medication”, and “postmenopausal women or men above age 50”) [44]. To this end, our models provide a simplified and specific way of predicting bone mass in PWH using only two or three variables, which are more practical, non-invasive, and accessible for low-and middle-income countries. Additionally, it is important to highlight that only quantitative measurement of bone mass cannot accurately predict fracture risk, and continuous patient monitoring is a safer alternative in fracture prevention. This underscores the combination of qualitative and quantitative measures as essential strategies in this scenario.

To the best of our knowledge, there are no anthropometric models to identify and monitor bone alterations in adults living with HIV. We believe that our study has clinical implications worldwide, particularly for individuals from low- and middle-income countries. Future studies should continue investigating body composition alterations in PWH and confirm the broad application of our findings.

## 5. Conclusions

Our anthropometric predictive models for BMC are valid, simplified, safe, and represent a low-cost alternative for monitoring bone health in PWH. Early identification of BMC reductions may allow timely prophylactic interventions for osteopenia and osteoporosis in PWH.

## Figures and Tables

**Table 1:** General sociodemographic, anthropometric, and body composition characteristics of the sample overall and separated by sex.

Variables	People Living with HIV						*p* Value
	Total (*n* = 104)		Male (*n* = 64)		Female (*n* = 40)		
	Mean (95% CI)	Min; Max	Mean (95% CI)	Min; Max	Mean (95% CI)	Min; Max	
Age (years)	44.85 (43.2; 46.5)	21.7; 58.8	45.34 (43.2; 47.5)	21.7; 58.8	44.03 (40.6; 46.9)	25.0; 58.8	0.399
Diagnosis of HIV (months)	116.31 (98.4; 131.1)	6.2; 376.5	118.67 (98.2; 137.8)	6.6; 278.1	112.21 (79.8; 140.5)	6.2; 376.5	0.523
Exposure to ART (months)	87.57 (74.6; 100.6)	6.2; 216.3	91.90 (75.5; 107.8)	6.2; 216.3	79.81 (58.8; 103.9)	6.2; 207.1	0.378
Protease inhibitors use (*n*/%)	45 (43.3)		30 (46.9)		15 (37.5)		
Formal education (years)	8.5 (7.9; 9.3)	2.0; 15.0	8.98 (8.0; 9.9)	2.0; 15.0	7.67 (6.7; 8.6)	4.0; 12.0	0.670
White (*n*/%)	68 (65.4)		44 (68.8)		24 (60.0)		
Black (*n*/%)	8 (7.7)		5 (7.8)		3 (7.5)		
Asian (*n*/%)	8 (7.7)		4 (6.3)		4 (10.0)		
Pardo (*n*/%)	20 (19.2)		11 (17.2)		9 (22.5)		
Height (m)	1.65 (1.6; 1.7)	1.45; 1.85	1.70 (1.7; 1.7)	1.5; 1.8	1.56 (1.5; 1.6)	1.4; 1.7	<0.001
Body weight (kg)	68.07 (65.7; 70.6)	50.0; 99.0	70.48 (68.3; 73.5)	48.0; 99.0	63.9 (59.4; 67.7)	50.0; 83.0	0.004
Body mass index (kg/m^2^)	24.85 (24.0; 25.6)	17.1; 34.8	24.14 (23.5; 25.0)	17.1; 31.9	26.09 (24.3; 27.5)	17.1; 34.8	0.042
Body composition by DXA							
Total-BMC (g)	2100.65 (2027.8; 2189.8)	1010.3; 3199.6	2234.84 (2154.4; 2322.4)	1291.9; 3199.6	1869.55 (1745.6; 1994.2)	1010.3; 2723.8	<0.001
Total-aBMD (g/cm^2^)	1.10 (1.1; 1.1)	0.8; 1.4	1.11 (1.1; 1.1)	0.8; 1.4	1.10 (1.1; 1.1)	0.9; 1.4	0.356
Anthropometric measurement							
Body circumferences (cm)							
Left arm extended	28.6 (27.7; 29.1)	15.0; 36.0	28.5 (28.0; 29.5)	15.0; 36.0	27.80 (26.5; 29.2)	19.2; 35.0	0.217
Left arm contracted	29.37 (28.7; 30.0)	20.0; 36.5	29.59 (28.9; 30.4)	21.9; 36.5	28.98 (27.3; 30.1)	20.0; 36.2	0.197
Left forearm	25.32 (24.9; 25.9)	18.7; 30.5	26.19 (25.8; 26.7)	22.2; 30.5	23.84 (23.1; 24.7)	18.7; 29.0	<0.001
Left wrist	16.25 (15.9; 16.7)	12.5; 19.5	16.64 (16.2; 17.3)	13.9; 19.5	15.58 (15.2; 16.1)	12.5; 18.0	0.009
Right arm extended	28.89 (28.2; 29.6)	19.6; 38.1	29.00 (28.3; 29.9)	22.5; 35.0	28.70 (27.2; 30.0)	19.6; 38.1	0.465
Right arm extended corrected	25.26 (24.8; 25.9)	18.1; 33.3	26.41 (25.9; 27.1)	21.7; 33.3	23.30 (22.5; 24.1)	18.1; 27.3	<0.001
Right arm contracted	29.87 (29.2; 30.6)	20.5; 38.0	30.10 (29.4; 30.9)	24.0; 36.0	29.49 (28.0; 30.8)	20.5; 38.0	0.270
Right arm contracted corrected	26.24 (25.7; 27.0)	19.1; 33.3	27.50 (27.0; 28.2)	22.7; 33;3	24.09 (23.3; 24.9)	19.1; 28.2	<0.001
Right forearm	25.81 (25.4; 26.3)	19.0; 30.5	26.71 (26.3; 27.2)	23.2; 30.5	24.26 (23.6; 25.1)	19.0; 30.0	<0.001
Right wrist	16.27 (16.0; 16.6)	12.0; 19.0	16.63 (16.4; 16.9)	14.3; 19.0	15.66 (15.2; 16.1)	12.0; 18.0	<0.001
Left thigh	51.19 (49.3; 53.1)	15.0; 72.3	49.68 (47.6; 51.7)	16.0; 61.3	53.81 (50.2; 57.3)	15.0; 72.3	0.039
Left medial calf	34.67 (34.0; 35.3)	24.2; 42.5	34.96 (34.4; 35.7)	29.5; 39.5	34.19 (32.8; 35.5)	24.2; 42.5	0.167
Left ankle	21.33 (21.0; 21.7)	17.0; 24.6	21.65 (21.3; 22.1)	19.4; 24.6	20.80 (20.3; 21.5)	17.0; 24;1	0.016
Right thigh	52.94 (51.6; 54.4)	36.0; 72.2	51.6 (50.4; 53.1)	39.5; 63.3	55.24 (52.7; 58.2)	36.0; 72.2	0.012
Right thigh corrected	47.85 (47.0; 48.9)	34.6; 60.2	47.93 (47.0; 49.1)	38.6; 60.2	47.72 (46.0; 49.7)	34.6; 57.8	0.848
Right medial calf	34.74 (34.0; 35.4)	25.0; 42.0	35.03 (34.4; 35.8)	29.0; 41.0	34.24 (32.8; 35.6)	25.0; 42.0	0.174
Right medial calf corrected	32.06 (31.0; 32.7)	22.3; 38.1	33.21 (32.7; 34.0)	27.9; 38.1	30.06 (28.9; 31.0)	22.3; 35.0	<0.001
Right ankle	21.29 (21.0; 21.7)	17.5; 25.0	21.67 (21.4; 22.1)	19.2; 25.0	20.65 (20.2; 21.2)	17.5; 24.0	0.002
Shoulder	105.52 (104.0; 107.2)	83.5; 120.5	108.23 (106.8; 110.0)	87.0; 120.5	100.88 (98.0; 103.2)	83.5; 112.0	<0.001
Breast	93.45 (91.7; 95.3)	75.0; 109.6	97.48 (96.0; 99.3)	82.5; 109.6	86.53 (83.7; 88.4)	75.0; 102.4	<0.001
Waist	86.77 (85.0; 88.7)	64.0; 105.0	88.40 (86.6; 90.9)	67.3; 105.0	83.93 (80.2; 86.8)	64.0; 98.3	0.007
Abdomen	90.69 (88.0; 93.2)	65.0; 111.0	89.70 (86.4; 93.0)	65.0; 110.4	92.33 (87.9; 95.6)	69.0; 111.0	0.511
Hip	93.82 (91.9; 95.6)	75.5; 117.4	91.66 (90.1; 93.7)	75.5; 112.8	97.55 (93.8; 101.1)	79.5; 117.4	0.004
Skinfold thickness (mm)							
Triceps	11.55 (9.8; 12.8)	1.0; 34.5	8.27 (7.2; 9.4)	1.0; 20.0	17.22 (13.9; 19.7)	4.1; 34.5	<0.001
Thigh	16.18 (13.8; 18.5)	3.0; 46.0	11.69 (9.7; 14.1)	3.0; 35.0	23.93 (20.0; 27.9)	4.2; 46.0	<0.001
Medial calf	8.5 (7.1; 9.9)	0.2; 28.0	5.77 (4.8; 7.1)	0.2; 26.0	13.3 (10.8; 16.2)	0.5; 28.0	<0.001

**Table 2:** Predictive models for bone mineral content (BMC) in 104 males and females living with HIV.

	Independent Variables							Validation	
**Models for Males**	**Body Weight (Kg)**	**BMI (kg/m^2^)**	**RAFCC** **(cm)**	**β**	**r^2^** **Adjust**	**SEE (g)**	**95 %CI**	**Q^2^** **_PRESS_**	**S** **_PRESS (g)_**
1	22.5 ± 2.6			642.72 ± 182.13	0.55	238.74	220.3 to 245.8	0.52	243.14
2	42.1 ± 4.9	−78.4 ± 17.4		1164.90 ± 196.78	0.67	208.74	201.6 to 232.8	0.64	215.07
3	40.1 ± 4.7	−86.9 ± 17.0	34.8 ± 13.6	557.55 ± 302.98	0.70	199.97	184.1 to 229.9	0.67	208.25
**Models for Females**	**Body weight (Kg)**	**AC (cm)**	**RAFC (cm)**	**β**	**r^2^** **adjust**	**SEE (g)**	**95 %CI**	**Q^2^** **_PRESS_**	**S** **_PRESS (g)_**
1	18.0 ± 4.1			679.85 ± 264.91	0.33	300.71	287.0 to 310.2	0.30	303.60
2	41.2 ± 7.3	−29.7 ± 8.2		1950.43 ± 418.87	0.50	255.89	233.5 to 266.6	0.47	260.55
3	58.9 ± 8.7	−34.8 ± 7.5	−53.5 ± 17.0	2881.94 ± 479.11	0.65	220.96	179.4 to 238.0	0.62	221.90

Note: HIV: Human immunodeficiency virus; Body weight (Kg): body weight in kilograms; BMI (kg/m^2^): body mass index (kilograms/meters square); RAFCC (cm): right arm flexed corrected circumference (centimeters); AC (cm): abdomen circumference (centimeters); RAFC (cm): right arm flexed circumference (centimeters); β: beta value; r^2^ adjust: r square adjusted; SEE (g): standard error of estimate in grams; CI 95%: confidence interval; Q^2^ _PRESS_: coefficient r square PRESS_;_ S _PRESS (g)_: error of estimate PRESS in grams.

## Data Availability

The dataset supporting the conclusions of this article is included in the article. Original data are available by request from the authors.
